# *Clock* gene variation in *Tachycineta* swallows

**DOI:** 10.1002/ece3.73

**Published:** 2012-01

**Authors:** Roi Dor, Caren B Cooper, Irby J Lovette, Viviana Massoni, Flor Bulit, Marcela Liljesthrom, David W Winkler

**Affiliations:** 1 Cornell Lab of Ornithology, Cornell UniversityIthaca, New York 14850; 2 Department of Ecology and Evolutionary Biology, Cornell UniversityIthaca, New York 14853; 3 Departamento de Ecología, Genética y Evolución, Facultad de Cs. Exactas y Naturales, Universidad de Buenos AiresCABA, C1428EGA, Argentina; 4 Centro Austral de Investigaciones CientíficasCADIC-CONICET, Bernardo Houssay 200, V9410BFD Ushuaia, Tierra del Fuego, Argentina

**Keywords:** Circadian, *Clock*, polyglutamine, *Tachycineta*, time of breeding, tree swallow

## Abstract

Many animals use photoperiod cues to synchronize reproduction with environmental conditions and thereby improve their reproductive success. The circadian clock, which creates endogenous behavioral and physiological rhythms typically entrained to photoperiod, is well characterized at the molecular level. Recent work provided evidence for an association between *Clock* poly-Q length polymorphism and latitude and, within a population, an association with the date of laying and the length of the incubation period. Despite relatively high overall breeding synchrony, the timing of clutch initiation has a large impact on the fitness of swallows in the genus *Tachycineta*. We compared length polymorphism in the *Clock* poly-Q region among five populations from five different *Tachycineta* species that breed across a hemisphere-wide latitudinal gradient (Fig. 1). *Clock* poly-Q variation was not associated with latitude; however, there was an association between *Clock* poly-Q allele diversity and the degree of clutch size decline within breeding seasons. We did not find evidence for an association between *Clock* poly-Q variation and date of clutch initiation in for any of the five *Tachycineta* species, nor did we found a relationship between incubation duration and *Clock* genotype. Thus, there is no general association between latitude, breeding phenology, and *Clock* polymorphism in this clade of closely related birds.

**Figure 1:**
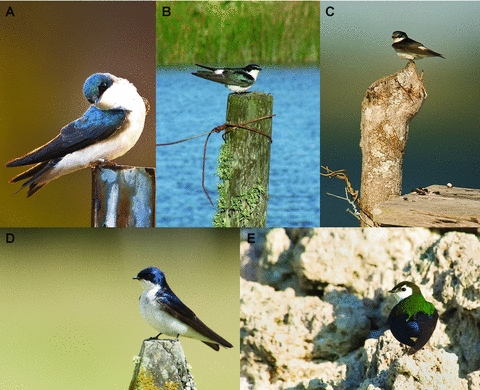
Photos of *Tachycineta* swallows that were used in this study: A) *T. bicolor* from Ithaca, New York, B) *T. leucorrhoa* from Chascomús, Argentina, C) *T. albilinea* from Hill Bank, Belize, D) *T. meyeni* from Puerto Varas, Chile, and E) *T. thalassina* from Mono Lake, California, Photographers: B: Valentina Ferretti; A, C-E: David Winkler.

Photos of *Tachycineta* swallows that were used in this study: A) *T. bicolor* from Ithaca, New York, B) *T. leucorrhoa* from Chascomús, Argentina, C) *T. albilinea* from Hill Bank, Belize, D) *T. meyeni* from Puerto Varas, Chile, and E) *T. thalassina* from Mono Lake, California, Photographers: B: Valentina Ferretti; A, C-E: David Winkler.

## Introduction

The phenology of reproduction has a critical influence on fitness in many animals. Thus, in order to optimize their reproductive effort in a seasonally varying environment, individuals have to estimate the best time for reproduction in advance by relying on cues from the changing environment. Most animals use photoperiod as the primary cue for their phenological timing (Saunders 1977; Aschoff 1981), and differences among individuals in the details of the physiological cue-response mechanism and how they fare in different environments are likely to have fitness consequences.

In birds, one of the most important and well-studied phenological traits is the date of clutch initiation. Selection on the timing of reproduction is expected to be stronger for species that usually raise only one brood per year, such as insectivorous birds that breed at higher latitudes with short seasonal reproductive windows and which rely on ephemeral resources (Charmantier et al. 2008) or have constraining molt schedules. Theory suggests that birds raising a single brood per year will be selected to lay at the time that is optimal for that one clutch, whereas birds raising multiple broods per year will lay at times that optimize their reproductive success over all of these clutches (Crick et al. 1993). Previous work on many bird species has shown that females that successfully time their reproductive effort produce more offspring (e.g., Perrins 1970; Verhulst and Tinbergen 1991; Sheldon et al. 2003; Charmantier et al. 2008). Interindividual variation in the time of breeding is often quite high, with some components of this variation having a heritable basis (Boag and van Noordwijk 1987; Svensson 1997; van der Jeugd and McCleery 2002; Sheldon et al. 2003). Thus variation in response to photoperiod cues will influence the timing of clutch initiation and therefore affect the individual's fitness.

Circadian rhythms, the endogenous ∼24-h biochemical, physiological, and behavioral cycles which exist in most organisms, are controlled by biological clocks that are well characterized genetically and biochemically (reviewed by Bell–Pedersen et al. 2005). Although endogenous, many clocks are entrained to, or synchronized by, photoperiodic zeitgebers (“time givers” or external time cues). Many of the genetic and biochemical aspects of the biological clock are shared among diverse taxa from insects to mammals (Panda et al. 2002), and genetic polymorphism in circadian clock genes has been associated with behavioral and ecological variation in many organisms (Katzenberg et al. 1998; Tauber and Kyriacou 2005; Johnsen et al. 2007; O’Malley and Banks 2008; Liedvogel et al. 2009). The vertebrate protein encoded by the circadian gene, *Clock*, is a transcription activator (as a heterodimer together with BMAL1) in the core circadian oscillator (Young and Kay 2001; Panda et al. 2002; Ko and Takahashi 2006). CLOCK protein sequence is -conserved across many avian taxa, yet considerable variation exists in the poly-Q (polyglutamine) repeat region (Fidler and Gwinner 2003) of this protein, in a region that in mammals (Avivi et al. 2001) and amphibians (Hayasaka et al. 2002) influences the transcription-activating potential of the heterodimer complex. In birds, cross-population analyses have shown a general association between *Clock* poly-Q allele length and latitude in the Blue Tit (*Cyanistes caeruleus*), but not in the Bluethroat (*Luscinia svecica*) (Johnsen et al. 2007).

Even though clutch initiation date is likely a quantitative trait influenced by many genes and their interactions with the environment, a recent within-population study in the Blue Tit showed that females with fewer poly-Q repeats bred earlier during the breeding season (Liedvogel et al. 2009). The same study found that females with fewer poly-Q repeats had shorter incubation periods, an intriguing pattern for which the authors had no functional explanation. The pace of embryonic development could be regulated by clock genes because embryos entrain to photoperiod (e.g., *Sturnus vulgaris*, Gwinner et al. 1997) and metabolic rate is greater in light than in dark (e.g., *Columba livia*, Prinzinger and Hinninger 1992; *Passer domesticus*, Cooper et al. 2011). However, similar analysis of a sympatric Great Tit (*Parus major*) population revealed low variability in *Clock* poly-Q and lack of association between either time of breeding or incubation duration and *Clock* poly-Q genotype (Liedvogel and Sheldon 2010). This evidence that *Clock* genetic variation influences avian reproductive timing in some but not all species suggests that the generality of this phenomenon should be further explored via similar investigations of other bird species.

In this study, we examined *Clock* poly-Q allelic length variation in populations of five of the nine species of swallows in the genus *Tachycineta*. *Tachycineta* swallows breed along an expansive latitudinal gradient in the Western Hemisphere, from the southern tip of South America to Alaska and northern Canada (Turner 2004). The populations studied here represent north-temperate, south-temperate, and tropical species that exhibit differences in many life-history traits that are likely influenced by the phenology of their annual cycles (Table 1). These species represent the two subclades within the monophyletic genus *Tachycineta* that are associated with geography: the North American/Caribbean clade (*T. bicolor* and *T. thalassina*) and a South/Central American clade (*T. albilinea*, *T. leucorrhoa*, and *T. meyeni*) (Whittingham et al. 2002; Cerasale et al. in review).

**Table 1:** Characteristics of the *Tachycineta* populations used in this study

Species	Population location	Breeding latitude	Breeding season	No. of broods	Migratory behavior
*T. bicolor*	Ithaca, NY	42°30′ N	May-Jul	1	Migratory
*T. thalassina*	Mono Lake, CA	38° N	Jun-Aug	1	Migratory
*T. albilinea*	Hill Bank, Belize	17°30′ N	Mar-Jun	2	Resident
*T. leucorrhoa*	Chascomús, Argentina	35°30′ S	Oct-Jan	2	Migratory
*T. meyeni*	Ushuaia, Argentina	55° S	Nov-Feb	2	Migratory

Our aims were to: (1) compare variation in *Clock* poly-Q region in *Tachycineta* swallows to variation reported in previous studies for other bird species, (2) test for a relationship between breeding latitude and *Clock* poly-Q variation, (3) test for a relationship between the seasonal decline in clutch size and *Clock* poly-Q allelic variation in order to examine the effect of selection pressure on clutch initiation date on *Clock* poly-Q variation, and (4) examine the relationship between *Clock* poly-Q variation and phenology of reproduction, as shown by timing of clutch initiation and incubation duration.

## Methods

### Study populations, breeding biology, and sample collection

We studied five populations from five species of *Tachycineta* swallows breeding in nest boxes: (1) *T. bicolor* from Ithaca, New York, USA, between 2002 and 2010; (2) *T. thalassina* from Mono Lake, California, USA, between 2008 and 2009; (3) *T. albilinea* from Hill Bank, Belize, in 2001, 2003, and 2009; (4) *T. leucorrhoa* from Chascomús, Argentina, in 2007 and 2008; and (5) *T. meyeni* from Ushuaia, Argentina, in 2006, 2008 and 2009. For each of these sites, these years represent the best combination of phenology data and blood samples obtained for large samples of individuals from the population (Table A1). We monitored the same populations in the multiple years.

In each of these colonies, we monitored nest boxes with repeated visits to record nest building and the dates of egg laying and hatching. Most of the adults were captured and uniquely marked with metal bands. Nestlings were measured and ringed between the ages of 6 and 12 days. Detailed information on the study populations and relevant field methods are included in Winkler and Allen (1996) and at http://golondrinas.cornell.edu. Blood samples were collected from adults and nestlings and were stored in lysis buffer until DNA was extracted.

For the association between lay date or incubation duration and *Clock* poly-Q variation, we limited our analyses only to breeding females, as there is no evidence that males strongly influence the timing of clutch initiation and because a previous study on Blue Tits (*C. caeruleus*) found an association between lay date and *Clock* poly-Q variation only for females (Liedvogel et al. 2009). We included only the first breeding attempt of the season in cases where females were known to have attempted multiple nestings. We measured the incubation period as the number of days from the laying of the last egg to the hatching of the first chick, excluding nest attempts for which either variable could not be estimated with ±1-day precision.

### Analysis of Clock poly-Q alleles

**Figure 2:**
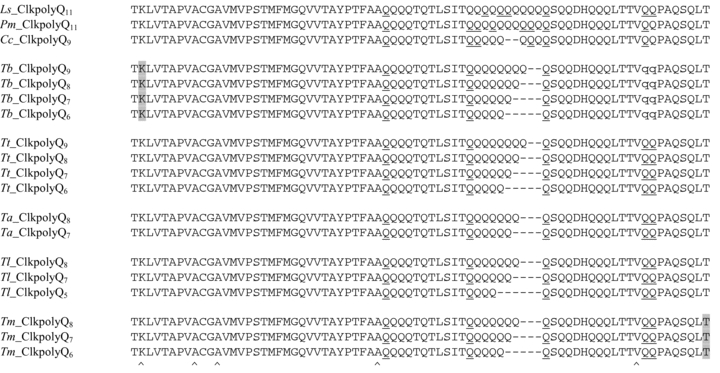
Amino acid alignment of *Clock* alleles from *Tachycineta* swallows (*T. bicolor*; *Tb*, *T. thalassina*; *Tt*, *T. albilinea*; *Ta*, *T. leucorrhoa*; *Tl*, *T. meyeni*; *Tm*) together with published Blue Tit (*Cyanistes caeruleus*; *Cc*), Great Tit (*Parus Major*; *Pm*), and Bluethroat (*Luscinia svecica*; *Ls*) alleles. For each sequence, the species name and number of *Clock* poly-Q repeats are shown. The predicted protein sequences of *Tachycineta Clock* poly-Q repeats only differ in the number of CAG codon (coded by Q) repeats (the first and last glutamine (Q) amino acids in the poly-Q repeat were coded by CAA codons). Q residues coded by CAA are underlined and lower-case Qs are within-population polymorphic sites encoded by either CAA or CAG. Caret symbol indicates synonymous substitutions (other than in glutamine) between the *Tachycineta* species in the flanking regions of the poly-Q repeat. Amino acids highlighted in gray represent synonymous substitutions between *Tachycineta* species.

Genomic DNA was extracted from blood using the E-Z 96 Tissue DNA kit (Omega Bio-Tek, Norcross, GA) or using the DNAeasy blood Extraction kit (Qiagen, Valencia, CA). To examine variability in *Tachycineta Clock* poly-Q region and verify the genetic sequence, we first amplified this region (corresponding to human *Clock* gene exon 20; Steeves et al. 1999) from four to eight individuals from each of the *Tachycineta* species using the sequencing primers developed by Johnsen et al. (2007). Ten-microliter polymerase chain reaction (PCR) amplifications included 10–100 ng DNA, 10 µM Tris-HCl, 50 µM KCl, 4 mM MgCl2, 0.25 mM of each nucleotide, 0.25 mM from each primer, and 0.025 U jumpstart Taq polymerase (Sigma-Aldrich, St. Louis, Missouri). PCR amplification conditions were: initial denaturation at 95°C for 4 min 30 sec; 30 cycles of denaturing at 95°C for 1 min, annealing at 64°C for 1 min, and extension at 72°C for 2 min, then a final extension at 72°C for 4 min 30 sec. PCR products were purified using Exonuclease and Shrimp Alkaline Phosphatase enzymatic reactions (United States Biochemical, Cleveland, OH). Purified products were cycle-sequenced in both directions using amplification primers and ABI BigDye Terminator chemistry. Sequencing products were cleaned using Sephadex columns and electrophoresed in an ABI 3730 Automated DNA Analyzer (Applied Biosystems, Foster City, CA). We aligned forward and reverse strands for each specimen and checked them using Sequencher 4.7 (Gene Codes Corp., Ann Arbor, MI). All sequence data are deposited in GenBank (Accession numbers JN896947-JN896985). The amplified sequence generated for all *Tachycineta* species matched the expected sequence for this gene in birds and was aligned to other avian species *Clock* gene in a BLAST search. We sequenced the *Clock* poly-Q region for 27 *Tachycineta* individuals. All *Tachycineta* sequences were identical to each other except for differences in their number of poly-Q repeats (Fig. 2). There were four synonymous differences between *Tachycineta* and Blue Tit sequences upstream and downstream of the poly-Q repeat. The first and last glutamine amino acids in the poly-Q repeat were coded by CAA codons, whereas the middle ones were coded exclusively by CAG codons. Thus, the only variation among the sequenced *Tachycineta* individuals was in the number of CAG codons.

All individuals were then screened for length polymorphism in the *Clock* poly-Q region using the genotyping primer set developed by Johnsen et al. (2007) in which the forward primer was labeled at the 5′ end with 6-FAM fluorescent dye. The PCR protocol was similar to the one used for sequencing (see above). PCR products were genotyped on an ABI 3100 Genetic Analyzer (Applied Biosystems) with GeneScan-500 LIZ (Applied Biosystems) as the molecular size standard. Allele sizes were estimated using Genemapper version 3.7 (Applied Biosystems) together with control samples with known repeat numbers determined by sequencing. We were able to successfully genotype all *Tachycineta* samples (*n* = 1016).

### Statistical analysis

Observed and expected heterozygosities for the *Clock* poly-Q region for each species were calculated using ARLEQUIN version 3.11 (Excoffier et al. 2005). We tested for departures from Hardy–Weinberg equilibrium (HWE) using GENEPOP version 4 (Raymond and Rousset 1995; Rousset 2008) with parameters of 10,000 dememorization, 10,000 batches, and 10,000 iterations. We used contingency tables to test whether allele frequencies were different between years for each species.

To test for a relationship between length polymorphism in the *Clock* poly-Q region and breeding phenology we used either mean *Clock* poly-Q allele size or the *Clock* poly-Q genotype. We examined the effect of *Clock* poly-Q region on lay date in separate analyses for each species using linear mixed models in which lay date was the dependent variable, female identity (band number) as a random effect to account for repeated measure for same females in different years, and breeding year and mean *Clock* poly-Q allele size (or *Clock* poly-Q genotype) as fixed effects. The effect of *Clock* poly-Q region on incubation duration was examined using linear mixed models in which incubation duration was the dependent variable, female's band number as a random effect and year, lay date, clutch size, and mean *Clock* poly-Q allele size (or *Clock* poly-Q genotype) as fixed effects. For *T. bicolor*, we also had information on female's age group (classified as “first year” or “after first year”) thus female's age was included as a fixed effect in all *T. bicolor* models. Data on incubation duration were not available for *T. meyeni*, therefore we were not able to examine the relationship between *Clock* poly-Q region and incubation duration for this species.

To examine fitness consequence on timing of breeding, we used generalized linear mixed models (SAS 9.1, SAS Institute Inc., Cary, NC) for the relationship between clutch size and lay date, with female's band number as a random effect and year and lay date as fixed effects (female's age was included as fixed effect in *T. bicolor* model). The effects (slopes) generated from this model of seasonal decline in clutch size were also used to examine the relationship between number of *Clock* poly-Q alleles and the effect of seasonal decline in clutch size using Spearman rank-order correlation. We used this test also to examine the correlation between population *Clock* poly-Q mean allele size and latitude.

## Results

### Clock poly-Q variation in *Tachycineta*

We genotyped a total of 1016 individuals from five *Tachycineta* species. Overall, we found five different length-variant alleles in *Tachycineta*, ClkpolyQ_5,6,7,8,9_, corresponding to 5–9 poly-Q repeats (Table 2). Maximum allelic polymorphism was four alleles (in *T. bicolor* and *T. thalassina*) and was as low as two alleles in *T. albilinea*, and the sample size ranged from 548 to 48 individuals per species. Observed heterozygosity ranged from 0.047 for *T. leucorrhoa* to 0.472 for *T. bicolor*. Genotype frequency in none of the five species deviated from HWE (all *P* > 0.05). In all species, one or two common alleles accounted for more than 90% of the allelic variation. We found no difference in genotype frequency between years for any of the species (Table A2).

**Table 2:** *Clock* poly-Q allele frequencies, number of individuals (*N*), number of alleles (*K*), mean allele size (with se), and observed heterozygosities (*H*) for the five species of *Tachycineta* used in this study. Allele frequencies did not deviate from Hardy–Weinberg equilibrium for any populations (all *P* > 0.05)

					Allele proportion					
						
Species	Latitude	*N*	*K*	Mean allele size (se)	Q_5_	Q_6_	Q_7_	Q_8_	Q_9_	*H*
*T. bicolor*	42°30′ N	548	4	8.27 (0.02)	0.000	0.020	0.014	0.640	0.326	0.472
*T. thalassina*	38° N	48	4	7.78 (0.05)	0.000	0.063	0.104	0.823	0.010	0.354
*T. albilinea*	17°30′ N	163	2	7.79 (0.02)	0.000	0.000	0.215	0.785	0.000	0.343
*T. leucorrhoa*	35°30′ S	169	3	7.01 (0.01)	0.006	0.000	0.970	0.024	0.000	0.047
*T. meyeni*	55° S	88	3	7.53 (0.04)	0.000	0.017	0.438	0.545	0.000	0.443

### *Clock* poly-Q and latitude

**Figure 3:**
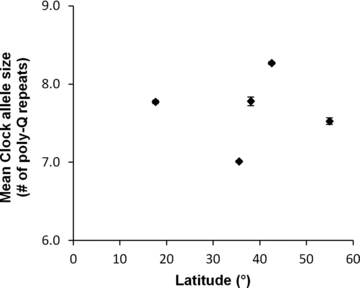
Relationship between mean *Clock* poly-Q allele size and breeding latitude in five populations of five species of *Tachycineta* swallows. Detailed data are provided in Table 2.

The association between population *Clock* poly-Q mean allele size and latitude was examined across the five *Tachycineta* species. Despite the wide range of variation in breeding latitudes, there was no correlation between breeding latitude and *Clock* poly-Q mean allele size (Fig. 3; *N* = 5, *r_s_* = 0.20, *t* = 0.35, *P* = 0.747).

### *Clock* genotype and reproductive phenology

In each of the five species, we examined the relationship between females’*Clock* poly-Q genotypes and two breeding phenology variables: lay date and incubation duration. We found no evidence for a relationship between *Clock* poly-Q and lay date for any of the five *Tachycineta* species (Table 3). Female's age had a strong effect on lay date in *T. bicolor* (there was no information on female's age for the other species) in which first-year females started laying later than older females.

**Table 3:** Relationship between lay date and *Clock* poly-Q average allele size for *Tachycineta* species. Linear mixed models included female's band number as a random effect and breeding year and mean *Clock* poly-Q allele size as fixed effects (Female's age was included as a fixed effect only for *T. bicolor* since this information was not available for the other species). Similar models with *Clock* poly-Q genotype as fixed effect generated quantitatively similar results (not presented)

Species	Covariable	Estimate (SE)	df	F	P-value
*T. bicolor*	Age		1, 697	108.92	<0.0001
	Year		7, 652	43.57	<0.0001
	*Clock* average allele size	0.494 (0.612)	1, 462	0.65	0.419
*T. thalassina*	Year		1, 32	5.06	0.031
	*Clock* average allele size	−0.287 (0.515)	1, 32	0.31	0.581
*T. albilinea*	Year		2, 69.1	9.04	<0.001
	*Clock* average allele size	0.622 (1.384)	1, 71.7	0.20	0.655
*T. leucorrhoa*	Year		1, 67	1.95	0.167
	*Clock* average allele size	3.810 (2.129)	1, 102	3.20	0.077
*T. meyeni*	Year		2, 54	15.65	<0.0001
	*Clock* average allele size	−0.210 (0.712)	1, 54	0.09	0.769

We did not find any relationship between female's *Clock* poly-Q and incubation duration for any of the *Tachycineta* species (Table 4; data on incubation duration was not available for *T. meyeni*). The effects of clutch size and lay date on incubation duration were not consistent across species. Similar models for either lay date or incubation duration with *Clock* poly-Q genotype instead of *Clock* poly-Q mean allele as fixed effect generated similar results (not presented).

**Table 4:** Relationship between incubation duration and *Clock* poly-Q average allele size for *Tachycineta* species. Linear mixed models included female's band number as a random effect and breeding year, lay date, clutch size, and mean *Clock* poly-Q allele size as fixed effects (female's age was included as a fixed effect only for *T. bicolor* since this information was not available for the other species). Similar models with *Clock* poly-Q genotype as fixed effect generated quantitatively similar results (not presented). Data on incubation duration were not available for *T. meyeni*

Species	Covariable	Estimate (SE)	df	*F*	*P*-value
*T. bicolor*	Age		1, 630	0.80	0.372
	Year		7, 559	17.64	<0.0001
	Lay date	−0.075 (0.008)	1, 635	80.38	<0.0001
	Clutch size	−0.518 (0.059)	1, 638	78.19	<0.0001
	*Clock* average allele size	0.114 (0.126)	1, 392	0.83	0.362
*T. thalassina*	Year		1, 9.91	15.21	0.003
	Lay date	−0.104 (0.042)	1, 29	6.11	0.020
	Clutch size	0.176 (0.255)	7, 30.9	0.48	0.495
	*Clock* average allele size	0.151 (0.143)	1, 23.8	1.12	0.300
*T. albilinea*	Year		2, 68.9	2.41	0.097
	Lay date	−0.019 (0.020)	1, 84.6	0.99	0.323
	Clutch size	0.052 (0.354)	1, 85	0.02	0.884
	*Clock* average allele size	0.138 (0.271)	1, 66.3	0.26	0.613
*T. leucorrhoa*	Year		1, 60.6	1.60	0.211
	Lay date	0.011 (0.010)	1, 101	1.27	0.262
	Clutch size	−0.407 (0.165)	1, 102	6.12	0.015
	*Clock* average allele size	−0.395 (0.225)	1, 100	3.08	0.082

### Seasonal effect on clutch size

In order to estimate the potential selection on timing of breeding, we examined the relationship between clutch size (a measure of potential reproductive success) and lay date for all *Tachycineta* species. We found a decrease in clutch size with the progress of the breeding season for all *Tachycineta* species (Table 5), however this trend was only marginally significant for *T. albilinea*.

**Table 5:** Relationship between clutch size and lay date for the *Tachycineta* species. Linear mixed models included female's band number as a random effect and breeding year and lay date as fixed effects (female's age was included as a fixed effect only for *T. bicolor* since this information was not available for the other species)

Species	Covariable	Estimate (SE)	df	*F*	*P*-value
*T. bicolor*	Age		1, 706	0.80	0.372
	Year		7, 706	2.27	0.028
	Lay date	−0.058 (0.006)	1, 706	98.63	<0.0001
*T. thalassina*	Year		1, 12.7	2.83	0.117
	Lay date	−0.064 (0.019)	1, 13.4	11.81	0.004
*T. albilinea*	Year		2, 58.4	1.42	0.250
	Lay date	−0.013 (0.007)	1, 98	3.71	0.057
*T. leucorrhoa*	Year		1, 56.9	1.25	0.269
	Lay date	−0.014 (0.006)	1, 103	5.56	0.020
*T. meyeni*	Year		2, 56.2	1.69	0.193
	Lay date	−0.023 (0.011)	1, 58.6	4.24	0.044

**Figure 4:**
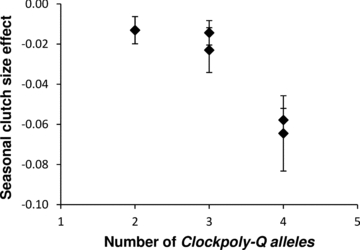
The effect of seasonal decline in clutch size (regression slope) and *Clock* poly-Q allelic diversity (number of alleles) in *Tachycineta*.

We have used the effect of seasonal decline in clutch size as an estimate of the intensity of selection on lay date and explore its relationship with *Clock* poly-Q allelic diversity. There was a significant relationship between the rate of seasonal decline in clutch size (the slope of the regression, presented in Table 5) and *Clock* poly-Q allelic diversity for *Tachycineta* (Fig. 4; *N* = 5, *r_s_* = −0.95, *t* = 5.20, *P* = 0.014).

## Discussion

The molecular genetic properties of the circadian clock have been well characterized for a wide range of organisms. Although most of the circadian genes are conserved even among diverse taxa, including birds, the poly-Q region of the circadian gene *Clock* exhibits both between-species and within-species variation along with within-individual heterozygosity in birds (Fidler and Gwinner 2003; Johnsen et al. 2007). This variation provides an opportunity to examine the direct relationship between genetic variation and life-history traits that may be associated with the circadian clock in wild bird populations. However, available results from the few wild bird populations that have been examined have not been conclusive (Johnsen et al. 2007; Liedvogel et al. 2009; Liedvogel and Sheldon 2010). Thus, the generality of the association between life-history traits and *Clock* poly-Q variation should be explored in other bird species. Swallows from the genus *Tachycineta* provide an excellent system to examine this relationship since they breed in a wide range of latitudes and exhibit variation in life-history traits that may be associated with the circadian clock.

Allelic variation in *Tachycineta* (Table 2) is lower than values reported previously for Blue Tit or Bluethroat (Johnsen et al. 2007), and more similar to the values reported for a Great Tit population (Liedvogel and Sheldon 2010), with shorter alleles compared to those of the other species (Q_5–9_ in *Tachycineta* compared to Q_9–17_ in Blue Tit, Q_11–15_ Great Tit, and Q_10–16_ in Bluethroat). Longer tandem repeats are more likely to be more polymorphic (Weber 1990; Edwards et al. 1991), therefore the lower variation in *Tachycineta* may be explained by their shorter *Clock* poly-Q repeats. However, polymorphism can be relatively low even in birds with long *Clock* poly-Q repeats (Liedvogel and Sheldon 2010).

Breeding latitudes of *Tachycineta* in our study ranged from 42° North to 55° South and included intervening tropical species. However, we were not able to detect any correlation between *Clock* poly-Q allele size and breeding latitude (Fig. 3). Unfortunately, we only had data on the *Clock* gene for five of nine *Tachycineta* species, thus data on the remaining species may increase our power to detect such a relationship. Each species in our study is represented by only one population. Therefore, this result could be due to either differences between the species or latitudinal differences, which cannot be distinguished. Therefore, more populations from each of the *Tachycineta* species should be sampled to examine the relationship between breeding latitude and *Clock* genotype. It is possible that this association is present only within-species and due to species differences, would not be observed even across closely related species.

Breeding phenology is important for reproductive success in most bird species including swallows (Stutchbury and Robertson 1988; Winkler and Allen 1996; Sheldon et al. 2003; Charmantier et al. 2008). In this study, the fitness consequences of breeding phenology are evident from the interaction between clutch size and date of clutch initiation, in which clutch size declines with the progress of the breeding season for *Tachycineta* species (Table 5). We failed to detect a pattern between *Clock* genotype and date of clutch initiation for females (Table 3). Unlike Blue Tits (Liedvogel et al. 2009), *Tachycineta* females with longer *Clock* poly-Q alleles did not initiate their clutches later. This is in accordance with the low variability of the *Clock* poly-Q in *Tachycineta* and similar to findings for a Great Tit population (Liedvogel and Sheldon 2010). Sample size for the *Tachycineta* species was very variable (Table A1), therefore it is possible that sample size limitations have prevented us from detecting a correlation between clutch initiation date and *Clock* genotype especially for species with the lower sample size. However, population sample size was not related to allele diversity (populations with highest and lowest sample sizes had the same number of alleles). Given the overall low variability of the *Clock* poly-Q in *Tachycineta* (even for *T. bicolor* with relatively high sample size), it is unlikely that we would have missed a pattern such as the one observed for the Blue Tit, in which the main effect was due to the more common alleles (Liedvogel et al. 2009).

Incubation duration is another feature of breeding phenology that we addressed in this study. Liedvogel et al. (2009) found an association between incubation duration and *Clock* poly-Q genotype, according to which incubation duration was shorter in both male and female Blue Tits with fewer *Clock* poly-Q repeats. We did not find any evidence for an association between incubation duration and *Clock* poly-Q genotype for *Tachycineta* females (Table 4). Currently, there is no a priori prediction for the relationship between *Clock* genotype and incubation duration, and the little data available do not allow conclusions about the functionality of this relationship. Further research is needed to examine this relationship and determine its generality in birds.

The relationship between *Clock* allele diversity and the slope of the seasonal decline in clutch size (Fig. 4) is counterintuitive, since, if the steep slope of clutch size with lay date suggests strong selection on lay date, then a greater number of alleles in the more strongly selected populations would be surprising. Alternatively, selection may actually act to increase polymorphism in photoperiodic response mechanisms in more variable environments nearer the poles, resulting in more *Clock* poly-Q alleles where clutch size-lay date slopes are steepest. It has been suggested that tandem-repeat length polymorphism, such as the *Clock* CAG repeat, may be selectively advantageous by itself (Wren et al. 2000; Johnsen et al. 2007). Therefore, it is possible that in cases where selection on clutch initiation date is stronger, for example, in populations that lay only one clutch per season such as *T. bicolor* and *T. thalassina* (Table 1), where reproductive success of early breeders is higher (Winkler and Allen 1996; Stutchbury and Robertson 1988), the population exhibits increased polymorphism in *Clock* poly-Q. This could be a result of between-year changes that shift the selection from year to year (change in “optimal” allele) or as a result of balancing selection on a suite of alleles (Wren et al. 2000). Of course, all of these selective interpretations must be weighed against a neutral hypothesis. In this case, the number of alleles may reflect the different effective population sizes of the species being compared, as *T. bicolor* and *T. thalassina* (the two species that have the most alleles) very likely have larger effective populations than do the other species. A more rigorous test of this hypothesis must await actual data on effective population sizes.

This study on *Clock* poly-Q variation in *Tachycineta* swallows increases the confusion regarding its association with life-history traits among avian taxa. *Tachycineta* swallows exhibit relatively low levels of *Clock* poly-Q variation, with no apparent correlation with latitude. Within-population analyses showed no evidence for a relationship between *Clock* poly-Q and clutch initiation date or between *Clock* poly-Q and incubation duration. Sample size varied between the *Tachycineta* populations included in this study, thus power differences might explain some of the negative results we obtained. However, the sample sizes used for these populations should have been large enough to capture the genetic variation in this locus, which was universally very low and therefore cannot account for the substantial differentiation among the species in these life-history traits. More generally, *Clock* poly-Q variation is diverse among bird species and accumulating data suggest that the association between *Clock* poly-Q variation and breeding latitude or within-population breeding phenology is probably not general for avian species.

It is interesting, however, that we have added a new member to the list of life-history features that can be influenced by *Clock* poly-Q variation in birds. This raises two interesting questions at the molecular genetic level: (1) why does each successive study seem to find an association of *Clock* poly-Q variation with a new phenotypic trait, and (2) why does every study of this system fail to replicate the patterns seen in other species? We suspect that the answer to the first question is merely that the circadian clock system in general is regulated and differentiates in both ecological and evolutionary time in very complicated ways. We have only begun to scratch the surface, and understanding this complexity will take many years of work. But there is no question that this genetic system is important and pervasive in its effects on reproductive biology.

One possible answer to the second question of the failure to find reported associations between *Clock* poly-Q and breeding phenotypes is that the variation in timing of breeding and incubation duration that are present in different species may be associated with variation in other genes in the circadian clock system. An investigation of variation of other loci that are part of the core circadian clock pathway, such as *Bmal1*, *Per*, and *Cry* (reviewed in Bell–Pedersen et al. 2005) might be interesting, however, given the conserved nature of these genes (Saleem et al. 2001; Fidler and Gwinner 2003), high levels of variation in coding regions of these other genes seems unlikely. Another possible answer would be that phenotypic variation in these traits is associated with molecular variation at different levels, including variation in expression patterns among species, populations, and individuals, and even among different organs within individuals (Yoshimura et al. 2000). It must be remembered in contemplating studies of gene expression patterns that the circadian clock system is highly variable in its levels of gene expression of the various genes throughout the day (Yoshimura et al. 2000), and patterns of gene expression are likely to be extremely time dependent. In contemplating gene expression studies, we have been at a loss to know how to standardize time of collection of samples in free-living populations for the appropriate comparisons among individuals and populations. Epigenetic effects, such as DNA methylation, provide additional levels of molecular variation that might be associated with phenotypic differentiation observed in life-history traits (reviewed in Jaenisch and Bird 2003).

These approaches will be challenging to implement for wild populations, and adequate methods should probably be developed first using model species, which allow controlled manipulations, behavioral experimentation in the laboratory, and captive breeding. The challenge then becomes to choose a model system with breeding biology that is controlled largely by the circadian system, as many model avian systems are derived from wild species that are probably controlled more by variations in food and rain than by photoperiod.

## Appendix

**Table A1:** Female sample sizes per year for each of the *Tachycineta* species used in this study for the association between *Clock* poly-Q length polymorphism and life-history traits (lay date or incubation duration)

Year	*T. bicolor*	*T. thalassina*	*T. albilinea*	*T. leucorrhoa*	*T. meyeni*
2001			29		
2002	112				
2003	92		36		
2004	34				
2005	76				
2006				52	6
2007	87			51	
2008	100	13			22
2009	111	19	32		27
2010	112				
Total observations	724	32	97	103	55
Total females	449	26	85	81	46

**Table A2:** Results for tests for among-year differences in *Clock* poly-Q allele frequencies for each of the *Tachycineta* species

Species	*χ^2^*	*df*	*P*-value
*T. bicolor*	20.97	21	0.461
*T. thalassina*	1.26	1	0.261
*T. albilinea*	1.40	2	0.497
*T. leucorrhoa*	0.186	1	0.667
*T. meyeni*	1.64	4	0.801
